# Inverse pH Gradient-Assay for Facile Characterization of Proton-Antiporters in *Xenopus* Oocytes

**DOI:** 10.3390/membranes14020039

**Published:** 2024-02-01

**Authors:** Zeinu Mussa Belew, Christa Kanstrup, Chengyao Hua, Christoph Crocoll, Hussam Hassan Nour-Eldin

**Affiliations:** DynaMo Center, Department of Plant and Environmental Sciences, Faculty of Science, University of Copenhagen, 1871 Frederiksberg C, Denmark; zmb@plen.ku.dk (Z.M.B.); cka@plen.ku.dk (C.K.); chcr@plen.ku.dk (C.C.)

**Keywords:** DTX/MATE, DTX18, proton-coupled antiporter, inverse pH-gradient transport assay, p-coumaroyl-agmatine transporter, *Xenopus laevis* oocytes

## Abstract

*Xenopus* oocytes represent one of the most versatile model systems for characterizing the properties of membrane transporters. However, for studying proton-coupled antiporters, the use of *Xenopus* oocytes has so far been limited to so-called injection-based transport assays. In such assays, where the compound is injected directly into the oocytes’ cytosol and transport is detected by monitoring substrate efflux, poor control over internal diffusion and concentration are incompatible with mechanistic characterizations. In this study, we present an inverse pH-gradient transport assay. Herein, an outward-facing proton gradient enables the characterization of proton antiporters via facile import-based transport assays. We describe two approaches for establishing sustained outward-facing proton gradients across the oocyte membrane, namely by applying alkaline external conditions or through surprisingly stable carbonyl cyanide m-chlorophenyl-hydrazone (CCCP)-mediated acidification of the cytosol. Previously, genetic evidence has shown that DTX18 from *Arabidopsis thaliana* is essential for the deposition of the hydroxycinnamic acid amide *p*-coumaroylagmatine (coumaroylagmatine) defence compound on the leaf surface. However, direct evidence for its ability to transport coumarol-agmatine has not been provided. Here, using *Xenopus* oocytes as expression hosts, we demonstrate DTX18’s ability to transport coumaroyl-agmatine via both injection-based and inverse pH-gradient transport assays. Notably, by showing that DTX18 is capable of accumulating its substrate against its concentration gradient, we showcase the compatibility of the latter with mechanistic investigations.

## 1. Introduction

In plants, members of the detoxification efflux carriers/multidrug and toxic compound extrusion (DTX/MATE) mediate secondary active transport of their substrate against the electrochemical gradient of protons across membranes [1]. Thus, characterizing such antiporters in heterologous systems is complicated by the need to establish a proton gradient in the opposite direction of the substrate concentration gradient. 

*Xenopus* oocytes represent a well-established model system for characterizing transport proteins. For characterizing proton-antiporters, substrates have so far been injected directly into the cytosol of the oocyte, and the oocyte has been incubated in an acidic external buffer. Transport activity has then been detected by monitoring a reduction in substrate concentration within the oocyte concomitant with an increase in external concentration [2,3,4]. This approach is suitable for qualitatively demonstrating transport activity and dependence on the proton gradient. However, due to the release of substrates into a large external volume, it is not well suited for characterizing whether the transporter functions as an active transporter. Moreover, due to the variability in oocyte size and substrate-dependent diffusion rate within the oocyte, this approach is not well suited for kinetic characterization, where precise control over concentration and assay time is needed. Accordingly, there is a need for easier and more versatile transport assays for characterizing proton-antiporters in *Xenopus* oocytes. 

In *Arabidopsis thaliana*, the deposition of coumaroylagmatine on leaf surfaces contributes to defence against *Phytophthora infestans* [5]. Genetic characterizations show that DTX18 is essential for the export of coumaroylagmatine [6], but until now, direct evidence for DTX18’s capability to transport coumaroylagmatine has not been provided.

In this study, we use *Xenopus* oocytes as expression hosts and establish a facile approach for both identifying substrates and characterizing the properties of DTX/MATE transporters. We use two different transport assays in *Xenopus* oocytes to demonstrate directly that DTX18 indeed does transport coumaroylagmatine and that it does so actively, depending on a proton gradient oriented in the opposite direction of the gradient of coumaroylagmatine. In the first assay, we use the “injection-based” export assays previously used to characterize other DTX members [2,3]. The second assay represents the main advance of this study. It is a so-called inversed pH-gradient transport assay, wherein we establish an outward-facing proton gradient via two different approaches. We show that DTX18-expressing oocytes incubated in alkaline external buffer or oocytes, wherein the cytosol has been acidified, import coumaroylagmatine. The inverse pH-gradient assay has a low background, allows characterization of the fundamental transport mechanism of DTX18 and lays the foundation for rapid screens to identify substrates of other DTX/MATE transporters using *Xenopus* oocytes as expression hosts. 

## 2. Materials and Methods

### 2.1. Cloning and In Vitro Transcription 

The coding DNA sequence of *DTX18* was cloned into the *Xenopus* expression vector pNB1u using the USER cloning technique as described previously [7]. The pHluorin2 coding sequence in the pNB1u vector was kindly provided by Prof. Dietmar Geiger, University of Würzburg. Linear DNA template for in vitro transcription was generated by PCR with pNB1u plasmid-specific primers (fw: 5′-AATTAACCCTCACTAAAGGGTTGTAATACGACTCACTATAGGG-3′ and rv: 5′-TTTTTTTTTTTTTTTTTTTTTTTTTTTTTATACTCAAGCTAGCCTCGAG-3′). Capped cRNA was in vitro synthesized using the mMessage mMachine T7 Kit (Invitrogen, Thermo Fisher Scientific, Waltham, MA, USA) following the manufacturer’s instructions, and the cRNA concentration was normalized to 500 ng µL^−1^.

### 2.2. Expression in Xenopus Oocytes

Defolliculated *X. laevis* oocytes, stage V–VI, were purchased from Ecocyte Bioscience (Dortmund, Germany). Oocytes were injected with 50.6 nl of *DTX18* cRNA (or nuclease-free water as a mock control) using a Drummond NANOJECT II (Drummond Scientific Company, Broomall, PA, USA). The oocytes were then incubated for three days at 16 °C in kulori buffer (90 mM NaCl, 1 mM KCl, 1 mM MgCl_2_, 1 mM CaCl_2_, and 10 mM HEPES at a pH of 7.4) supplemented with 100 µg mL^−1^ amikacin.

### 2.3. Transport Assays in X. laevis Oocytes

#### 2.3.1. Injection-Based Export Assay

Three days after cRNA injection, oocytes were injected with 23 nL of 10 mM coumaroylagmatine (synthesized by Smolecule, San Antonio, TX, USA). For time = 0 samples, coumaroylagmatine-injected oocytes were directly transferred to Eppendorf tubes and then homogenized with 50% methanol; the rest of the injected oocytes were incubated in a 96 well U-bottom microtiter plate (Greiner Bio-One, Merck Life Sciences, Søborg, Denmark) with three oocytes per well containing 150 µL kulori buffer at a pH of 5.0 or a pH of 7.4 for 150 min. Then, 150 min after incubation, oocytes and the assay buffer were collected separately. For the assay buffer samples, 15 µL of the external buffer was sampled prior to harvesting the oocytes and then mixed with 50 µL 50% methanol. The oocytes were harvested, washed four times in kulori buffer and then homogenized with 50% methanol. The homogenate was centrifuged at 15,000× *g* at 4 °C for 15 min, and then the supernatant was diluted three times with water and filtered through a 0.22 µm filter plate (MSGVN2250, Merck Millipore, Darmstadt, Germany). Samples were analysed by analytical liquid chromatography coupled to mass spectrometry (LC-MS/MS) for coumaroylagmatine quantification as described below.

#### 2.3.2. Uptake Assays

Uptake assays were carried out as described previously [8] with some modifications. The assays were performed in kulori buffer (90 mM NaCl, 1 mM KCl, 1 mM MgCl_2_, and 1 mM CaCl_2_) at a pH of 5.0, a pH of 7.4, or a pH of 9.0 with 10 mM of MES, HEPES, or AMPSO as buffering agents, respectively. Three days after cRNA injection, oocytes were pre-incubated in kulori buffer at a pH of 5.0, a pH of 7.4, or a pH of 9.0 for five minutes. When CCCP was used for acidification of intracellular oocyte pH, oocytes were pre-incubated in 100 µM CCCP in kulori buffer with a pH of 6.5 for 45 min. After pre-incubation, oocytes were incubated in 0.5 mL of kulori buffer of respective pH containing 50 or 75 µM coumaroylagmatine (synthesized by Smolecule, San Antonio, TX, USA) for 60 min (except for the time-course assay). Subsequently, oocytes were washed four times in kulori buffer of the respective pH, homogenized with 50% methanol and then stored at −20 °C overnight. The homogenate was centrifuged at 15,000× *g* at 4 °C for 15 min, and then the supernatant was diluted three times with water and filtered through a 0.22 µm filter plate (MSGVN2250, Merck Millipore). Samples were analysed using LC-MS/MS for coumaroylagmatine quantification as described below.

### 2.4. Intracellular Acidification and pH Measurement in Xenopus Oocytes

The cytosolic pHluorin2 protein was expressed in the oocytes as described above. Single oocytes expressing pHluorin2 were transferred to a 96 well conical bottom plate (Thermo Fisher Scientific, Waltham, MA, USA), positioning the vegetal hemisphere of the oocyte facing upward as much as possible. Initially, the oocytes were incubated in 150 µL of kulori buffer at a pH of 7.4 for 15 min. Subsequently, the buffer was replaced with kulori buffer with a pH of 6.5 containing either 100 µM CCCP or 30 mM acetic acid. After a 45 min incubation period, the buffer was switched to either a pH of 7.4 or a pH of 9.0 kulori buffer.

During the incubation of oocytes in each buffer, pH measurements were conducted by illuminating the oocytes at 395 and 475 nm, and the fluorescence emitted at 509 nm was recorded using a microplate reader (BioTek Synergy H1, Agilent, Santa Clara, CA, USA). The relative intracellular pH was expressed through the ratio of emissions at 395 and 475 nm excitation (395/475 nm). A decline in this ratio signified a reduction in cytosolic pH.

### 2.5. LC-MS/MS Analysis

Coumaroylagmatine in the diluted oocyte extracts was subjected to analysis by liquid chromatography coupled to mass spectrometry. Chromatography was performed on an Advance UHPLC system (Bruker, Bremen, Germany). Separation was achieved on a Kinetex 1.7u XB-C18 column (100 × 2.1 mm, 1.7 μm, 100 Å, Phenomenex, Torrance, CA, USA). Formic acid (0.05%, *v*/*v*) in water and acetonitrile (supplied with 0.05% formic acid, *v*/*v*) were employed as mobile phases A and B, respectively. The elution profile was: 0–0.1 min, 5% B; 0.1–1.0 min, 5–45% B; 1.0–3.0 min, 45–100% B; 3.0–3.5 min, 100% B; 3.5–3.55 min, 100–5% B and 3.55–4.7 min, 5% B. The mobile phase flow rate was 400 μL min^−1^. The column temperature was maintained at 40 °C. The liquid chromatography was coupled to an EVOQ Elite TripleQuad mass spectrometer (Bruker, Bremen, Germany) equipped with an electrospray ion source (ESI) operated in positive and negative ionization modes. The instrument parameters were optimized by infusion experiments with pure standards. The ion spray voltage was maintained at +5000 V and −3000 V in positive and negative ion modes, respectively. The cone temperature was set to 350 °C, and the cone gas was set to 20 psi. The heated probe temperature was set to 250 °C, and the probe gas flow was set to 50 psi. Nebulizing gas was set to 60 psi, and collision gas was set to 1.6 mTorr. Nitrogen was used as a probe and nebulizing gas, and argon was used as a collision gas. The active exhaust was constantly on. Multiple reaction monitoring (MRM) was used to monitor analyte precursor ion → fragment ion transitions. MRM transitions and collision energies were optimized by direct infusion experiments into the MS source. Detailed values for mass transitions are listed in Table 1. Both Q1 and Q3 quadrupoles were maintained at unit resolution. Bruker MS Workstation software (Version 8.2.1, Bruker, Bremen, Germany) was used for data acquisition and processing. Linearity in ionization efficiencies was verified by analysing a dilution series of the standard. Quantification of coumaroylagmatine was achieved using an external standard curve diluted with the same oocyte matrix as the actual samples.

## 3. Results

### 3.1. DTX18 Transports Coumaroylagmatine in Xenopus Oocytes

**Figure 1:**
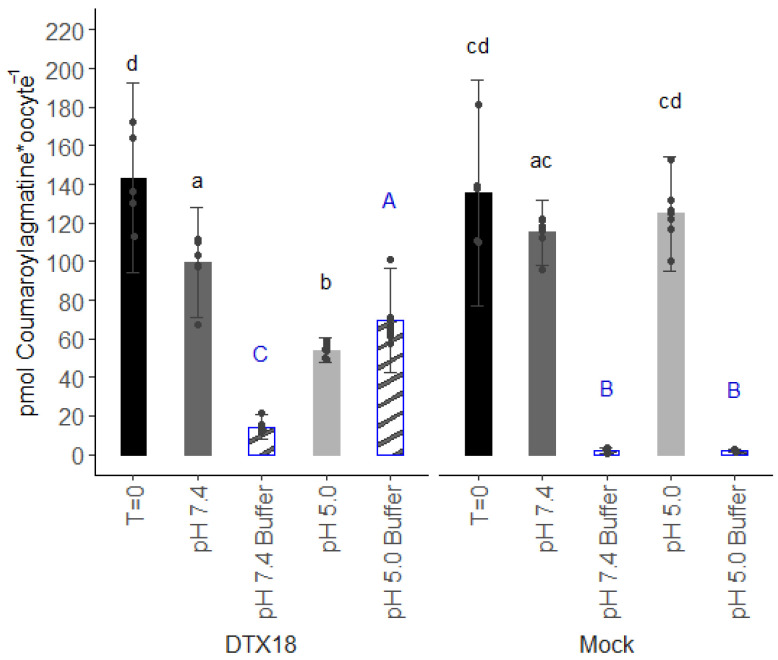
Export of coumaroylagmatine by DTX18 in *X. laevis* oocytes. Coumaroylagmatine was directly injected into DTX18-expressing or mock (water-injected) oocytes. The coumaroylagmatine content inside the oocyte and in the assay buffer was analysed after 150 min of incubation at a pH of 5.0 or 7.4. T = 0 (black bars) are from oocytes harvested right after coumaroylagmatine injection. Full grey bars are oocyte samples after 150 min; striped bars with blue borders are assay buffer samples after 150 min. Accumulated coumaroylagmatine in oocytes and assay buffer were quantified by LC-MS/MS analyses. The *Y*-axis represents the amount of coumaroylagmatine retained in a single oocyte or exported to assay buffer from a single oocyte. Error bars represent ± s.d., n = 5–8. Statistical analysis was performed with a nested ANOVA and Tukey posthoc test (*p* < 0.05) within a subset of the data (oocyte/buffer samples) on the model; Concentration_coumaroylagmatine~pH/RNA. Letters indicate statistically significant differences. Small black letters are for oocyte samples, and capital blue letters are for buffer samples. ANOVA tables are in Supplementary Tables S1 and S2.

To test the ability of DTX18 to transport coumaroylagmatine, we used *Xenopus* oocytes as a heterologous expression system. DTX18-expressing oocytes were injected with coumaroylagmatine and assayed for export of coumaroylagmatine into the external buffer. Compared to initial injected amounts (T = 0), the content of coumaroylagmatine in DTX18-expressing oocytes decreased by approximately 30% 150 min after injection when incubated in a buffer at an external pH of 7.4. Corresponding amounts of coumaroylagmatine accumulated in the external assay buffer. In mock oocytes, no significant reduction was observed in internal coumaroylagmatine content throughout the assay (Figure 1). When the coumaroylagmatine-injected oocytes were incubated at a pH of 5.0, the amount of coumaroylagmatine inside DTX18-expressing oocytes was reduced by approximately 60%, and the increase in the assay buffer was significantly higher than when the oocytes had been incubated at a pH of 7.4 (Figure 1). In contrast, mock oocytes did not show significant changes in the coumaroylagmatine level. These transport assays show that DTX18 can indeed transport coumaroylagmatine and that its transport activity increases in the presence of an opposing pH gradient across the membrane.

### 3.2. Reversing the Direction of DTX18-Mediated Coumaroylagmatine Transport

**Figure 2:**
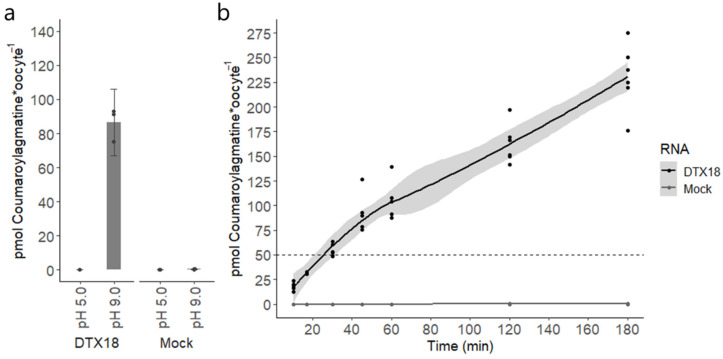
Uptake of coumaroylagmatine by DTX18 in *Xenopus* oocytes. (**a**) DTX18-expressing or mock (water-injected) oocytes were incubated in 75 μM coumaroylagmatine-containing buffer at a pH of 5.0 or a pH of 9.0 for 60 min. The dashed line represents the compound concentration in the buffer. Accumulated coumaroylagmatine in oocytes was quantified by LC-MS/MS analyses. Error bars represent ± s.d. n = 4 (4 × 4 oocytes). (**b**) Time-dependent accumulation of coumaroylagmatine relative to the assay buffer concentration in DTX18-expressing oocytes. DTX18-expressing or mock (water-injected) oocytes were incubated in a buffer with a pH of 9.0 containing 50 μM coumaroylagmatine and analysed at 7 different time points (ranging from 10 min to 180 min). The model for the line between the seven time points is a local regression fitting (loess method, y~x), and the grey area surrounding the line shows a confidence interval of 0.95. Accumulated coumaroylagmatine in oocytes was quantified by LC-MS/MS analyses. n = 8.

Next, we explored if we could characterize DTX18 via an assay, wherein the substrate is supplied to the external buffer and transport activity is monitored by detecting the import of the substrate. This requires a reversal of the transport direction by reversing the direction of the proton gradient across the oocyte membrane. Thus, an uptake assay was performed, wherein oocytes were incubated in 75 µM coumaroylagmatine-containing buffer at a pH of 9.0 (pH gradient oriented in opposite direction to substrate concentration gradient) and as a control at a pH of 5.0 (pH gradient oriented along substrate concentration gradient). DTX18-expressing oocytes did not accumulate coumaroylagamatine after 60 min of incubation at a pH of 5.0 (Figure 2a). However, at a pH of 9.0, coumaroylagmatine accumulated slightly above the external buffer concentration in DTX18-expressing oocytes (Figure 2a). Mock (water-injected) oocytes did not accumulate detectable levels of coumaroylagmatine at either pH. This shows that the DTX18-mediated coumaroylagmatine transport direction can be reversed when the direction of the proton gradient across the oocyte membrane is reversed.

To determine whether DTX18-mediated coumaroylagmatine transport proceeds via an active transport mechanism, we conducted a time-course uptake assay. DTX18-expressing oocytes were incubated in a buffer at a pH of 9.0 containing 50 µM of coumaroylagmatine, and the accumulation of coumaroylagmatine was analysed over time (10–180 min). Mock oocytes did not accumulate detectable levels of coumaroylagmatine at any time point. In contrast, DTX18 showed a time-dependent uptake of coumaroylagmatine into oocytes, where the intracellular concentration of coumaroylagmatine reached that of the assay buffer concentration after 30 min of incubation and reached up to fourfold that of the assay buffer concentration after 180 min of incubation (Figure 2b).

### 3.3. DTX18 Transports Coumaroylagmatine in a Proton-Coupled Antiport Mechanism

**Figure 3:**
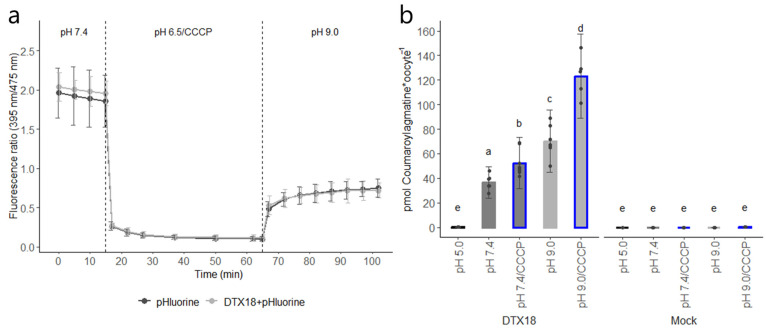
Inverse pH transport assay in *Xenopus* oocytes. (**a**) Intracellular acidification of *Xenopus* oocytes by CCCP. The ratio between the emission at 395 and 475 nm (395/475 nm) represents the relative intracellular pH. Relative cytosolic pH was represented by a dot, which represents the mean, and error bars represent ± s.d. (**b**) DTX18-expressing or mock (water-injected) oocytes were incubated in 50 μM coumaroylagmatine-containing buffer at different pH values (pH values of 5.0, 7.4 and 9.0) for 60 min. When it is applicable (blue border around the bar), 100 μM CCCP was used for preincubation at a pH of 6.5 for 45 min. Accumulated coumaroylagmatine in oocytes was quantified by LC-MS/MS analyses. Error bars represent ± s.d. n = 8. Statistical analysis was performed with a nested ANOVA and Tukey posthoc test (*p* < 0.05) on the model; Concentration_coumaroylagmatine~pH/RNA. Letters indicate statistically significant differences. ANOVA table in Supplementary Table S3.

The pHluorin2 protein is a ratiometric pH-sensitive green fluorescent protein [9]. pHluorin2 exhibits a bimodal excitation spectrum with two major peaks at 395 and 475 nm. Upon acidification, the fluorescence at 395 nm excitation decreases, accompanied by an increase at 475 nm [9]. We monitored CCCP- or acetate-induced cellular acidification over time in oocytes expressing pHluorin2 using a microplate reader. Incubating the oocytes in a buffer at a pH of 6.5 containing 100 μM CCCP or 30 mM acetic acid led to a gradual decrease in the 395/475 nm excitation ratio over time, indicating cytosolic acidification (Figure 3a and Supplementary Figure S1). Notably, compared to acetate, CCCP induced a sustained acidification of the cytosol of oocytes that persisted even after the CCCP was removed and the oocyte was moved to an external buffer with a neutral pH or a pH of 9.0 (Figure 3a and Supplementary Figure S1).

The ability of DTX18 to import coumaroylagmatine at a pH of 9.0 to fourfold the external buffer concentration suggests an active proton-coupled anti-port transport mechanism. To provide additional evidence for this, DTX18-expressing oocytes were incubated for 60 min in 50 µM coumaroylagmatine at different external pH values with and without prolonged preincubation in CCCP.

As seen before, no import activity was detected at an external pH of 5.0, whereas DTX18 imported up to 36 pmol of coumaroylagmatine when the external pH was increased to a pH of 7.4 (Figure 3b). The transport activity was significantly increased to approximately 66 pmol when the pH was increased from 7.4 to 9.0 (Figure 3b). DTX18-expressing oocytes whose cytosol had been acidified through pre-incubation in CCCP-containing buffer at a pH of 6.5, followed by incubation in either a pH of 7.4 or a pH of 9.0 coumaroylagmatine-containing buffers, accumulated significantly higher levels compared to the respective pH of 7.4 and pH of 9.0 assays (Figure 3b).

## 4. Discussion

In the present work, we describe a simple technique for characterizing proton:antiporter transporters in *Xenopus* oocytes. It is based on creating an outward-facing proton gradient that allows the characterization of proton:antiporters via an import-based transport assay. Albeit shown in other systems [10], the idea of reversing the pH gradient by incubating the cell in an alkaline buffer has not previously been tested in *Xenopus* oocytes.

Transient alkalization of oocyte cytosol is a well-known phenomenon that occurs during the process of hormone-induced oocyte maturation [11]. Interestingly, cytosolic alkalization via the external application of membrane-permeating weak organic bases has been shown to slowly induce maturation over the course of nine hours post-exposure (white dot formation associated with the breakdown of germinal vesicles) [11].

The AMPSO buffer used here to establish alkaline external pH belongs to the family of zwitterionic N-substituted aminosulphonic acids typically used in biological studies due to their low membrane permeation [12,13]. Upon exposure to a pH of 9.0, the ratiometric fluorescence emission of pHluorin2 indicated a slight alkaline shift in cytosolic pH that was reversed when reverting to an external pH of 5.5 (Supplementary Figure S2). However, we did not observe any visible white dot formation in the time-course experiment where mock and DTX18-expressing oocytes were incubated at a pH of 9.0 for 3 h. Moreover, in preliminary experiments, we saw no membrane permeation of two control compounds that do not normally permeate the membrane, namely 4-methylthiobutyl glucosinolate and esculin [14,15]. Thus, this mode of establishing an alkaline external pH is unlikely to lead to adverse effects on oocyte membrane integrity. It should be noted that several buffers with different properties can be used to establish an alkaline external pH. Attention should be paid to their ability to interfere with the assay. For example, propanol-derived buffers such as AMPD, AMP and AMPSO should be avoided if characterizing transporters of divalent cations are shown to interact with the buffer molecule [12].

The large size of the *Xenopus* oocyte makes it exceptionally well suited for the introduction, by microinjection, of virtually any molecule [16], including potential substrates of proton antiporters. Indeed, as shown here, DTX18’s capability to transport coumaroylagmatine was demonstrated by first injecting the substrate into the DTX18-expressing oocytes and subsequently monitoring reductions in intracellular content coinciding with external accumulation. While this assay is suitable for providing qualitative evidence for transport activity, our experience over the years indicates that this approach is not well suited for detailed mechanistic and kinetic investigations. For example, compounds injected into the oocyte must first traverse a distance within the cytosol before reaching the inner side of the membrane. This lag, which in our experience can vary in length depending on the physicochemical properties of the injected compound, complicates estimations of the initial transport rates required for kinetic characterizations. Moreover, as the interior of an oocyte has a finite volume, “over time” transport activity will deplete the internal content, which complicates accurate estimations of dose-dependent transport rates. Additionally, as the oocyte invariably exports into a much larger external volume, it is nearly impossible to deduce whether a given transporter is capable of transporting its substrate actively against a concentration gradient. Lastly, the injection-based export assays are technically demanding to perform. Especially the propensity of the injection needle to accumulate cell “goo” around the edges of the needle leads to varying diameters of the injection hole. This is especially an issue when conducting larger experiments where the needle may rest for longer periods “in the air” while one transfers oocytes, changes solutions, etc. As exemplified by the variation in the initial injected amounts (T = 0, Figure 1), variations in the injection hole affect the amount of injected substrate that leaks out immediately following injection. Thus, injection-based export assays display larger variation and pose a demand for oocytes of higher quality than when performing import-based assays where a second injection is not needed.

These drawbacks prompted us to test the inverse pH gradient idea in oocytes. Using the conventional injection-based export assay, our data provided the first direct evidence for DTX18’s capability to transport coumaroylagmatine and that the transport activity was affected by the amplitude of the opposing pH gradient (Figure 1). On the other hand, the inverse pH gradient approach provided several advantages. Foremost, we could now characterize the transport of coumaroylagmatine based on accumulation rather than reduction (Figure 2a). This enabled us to perform a time-resolved transport assay that showed DTX18’s capability to transport coumaroylagmatine against its concentration gradient (Figure 2b). This shows that DTX18 is a secondary active transporter that functions as a proton-coupled antiporter. These data also show that it is possible to control the direction of the transporter via the proton gradient. Future studies testing other members will reveal whether this is a common feature in the DTX/MATE family.

The basal internal pH of *Xenopus* oocytes typically lies in the range of ~7.4–7.8 [11]. Hence, we were surprised to see significant transport activity in both the injection-based and the reverse pH import assays when oocytes were incubated at an external pH of 7.4, which was expected to abolish the pH gradient across the membrane (Figure 1 and Figure 3b). Notably, no transport was observed when a large pH gradient was oriented in the same direction as the gradient of coumaroylagmatine (Figure 2a and Figure 3b). Although we cannot exclude that small pH gradients may have driven transport in these assays, our data indicate that in the absence of a proton gradient, the direction of DTX18-mediated transport is controlled by the concentration gradient of DTX18’s substrate.

Such “substrate-driven” transport activity has been described for other secondary active transporters. For example, the ZmSUT1 high-capacity sucrose:proton symporter from maize was shown to be able to reverse transport direction dependent on the sum of the sucrose gradient and the proton motive force. When the sucrose gradient dominates over the proton gradient and membrane potential, sucrose gradient-driven efflux of protons against the proton gradient could be demonstrated using the patch clamp technique [17]. Limited availability of coumaroylagmatine prevented us from investigating whether the DTX18-mediated transport of coumaroylagmatine was accompanied by a counterflow of protons and whether the transport at an external pH of 7.4 occurs with or without coupling to a proton.

To provide additional support for the proposed coumaroylagmatine:proton antiport mechanism, we resorted to reducing the oocytes’ internal pH. If DTX18 was indeed proton-coupled, then the import of coumaroylagmatine from both an external pH of 7.4 and a pH of 9.0 should increase if the internal pH was acidified.

CCCP is a protonophore that is typically used to dissipate the proton gradient across the plasma membrane. However, we showed that prolonged incubation in CCCP in acidic buffer can also be used to establish sustained acidification (>1 h) of the oocyte cytosol even after CCCP was removed and the oocytes were moved to an external neutral pH or a pH of 9.0 (Figure 3a and Supplementary Figure S1). In contrast, cytosolic acidification obtained via incubation in acetic acid was transient and rapidly returned to normal levels within ~20 min (Supplementary Figure S1). A possible explanation for CCCPs sustained acidification may be that they have reached the mitochondria of the oocytes, where it is also known to dissipate the proton gradient and mediate proton release from there (e.g., [18]). Having sustained acidification of the cytosol independent of external media composition opens the possibility for a range of experiments that would be difficult to perform within the limited time window provided by acetic acid-mediated acidification. Indeed, the significant increases in the amount of coumaroylagmatine accumulating in acidified oocytes provide additional support for DTX18 functioning as a proton-coupled antiporter (Figure 3b). More direct evidence for this can be provided by measuring alterations in the intracellular pH directly using intracellular pH electrodes or indirectly by monitoring the fluorescence of pHluorin2 through confocal microscopy [19]. In this study, we monitored the fluorescence of pHluorin2 via a fluorescence plate reader. Using this, we could not detect significant differences in the alkalization of oocyte cytosol in DTX18-expressing oocytes compared to mock when exposed to substrate (Supplementary Figure S3). This may be because we used a soluble pHluorin2 protein instead of fusing it to the transporter to measure more local pH changes in the near vicinity of the transporter. Additionally, in this study, DTX18 transport was detected via LCMS analyses. An alternative route could be to optimize the pHluorine2 pH sensor to detect substrate-induced activity.

## 5. Conclusions

In conclusion, we show that *Xenopus* oocytes can sustain prolonged incubation at an external alkaline pH. Together with the conventional injection-based export assay, this outward-facing pH gradient enabled us to provide direct evidence for DTX18’s ability to transport coumaroylagmatine and show that it is a secondary active transporter that likely utilizes a proton-coupled antiport mechanism to drive the transport of its substrate. Accordingly, this study lays the foundation for facile screening and characterization of members of transporter families functioning via a proton-coupled antiport mechanism.

**Figure 4:**
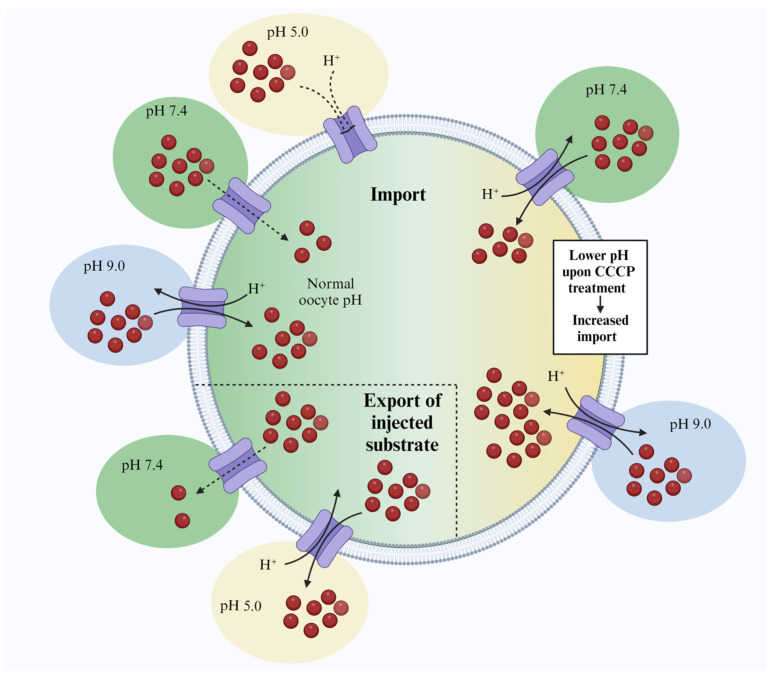
Representation of transport assays. Illustration of the different conditions applied in the various transport assays conducted in this study. Green indicates a neutral pH, blue indicates an alkaline pH and yellow indicates an acidic pH. A dotted rectangular section delineates the scenario where substrate has been injected into the oocyte and transport activity is detected by monitoring the reduction in content. Dotted arrows indicate weak transport compared to full arrows. Incomplete arrows indicate no detection of transport.

Additionally, we show that prolonged pre-incubation in CCCP-containing acidic buffer augmented the transport activity. Thus, CCCP provides an alternative approach to imposing an outward-facing proton gradient even at an external neutral pH. This may be useful for characterizing transporters of substrates that may be unstable at alkaline pH. The two approaches developed in this study for characterizing proton-coupled antiporters in *Xenopus* oocytes are summarized in Figure 4.

## Figures and Tables

**Table 1:** MRM transitions for LC-MS/MS analysis.

Analyte	Retention Time [min]	Q1 [*m*/*z*]	Q3 [*m*/*z*]	CE [eV]
Coumaroylagmatine [M + H]^+^	1.34	277.2	147.0 ^Qt^	21
		277.2	114.1	14
		277.2	218.1	15

Qt = quantifier ion, additional transitions were used for identification only; CE = collision energy; Q = quadrupole.

## Data Availability

The data presented in this study are available in supplementary material.

## Supplementary Materials

The following supporting information can be downloaded at: https://www.mdpi.com/article/10.3390/membranes14020039/s1, Figure S1: pHlourin2 ratio indicating cytosolic acidification by acetic acid or CCCP; Figure S2: Intracellular alkalization of *Xenopus* oocytes by application of buffer with pH 9.0; Table S1: ANOVA table Figure 1, oocyte samples; Table S2: ANOVA table Figure 1, buffer samples; Table S3: ANOVA table Figure 3b; Oocyte_raw data.xlsx.
