# Supplementary material for: Inverse pH Gradient-Assay for Facile Characterization of Proton-Antiporters in Xenopus Oocytes
Source: Membranes (Basel). 2024 Feb 1;14(2):39. doi: 10.3390/membranes14020039 (PMC10889953; doi:10.3390/membranes14020039)
Supplement: Supplementary file 1 [file membranes-14-00039-s001.zip › supplementary_figAndTable.pdf]

Supplementary Materials

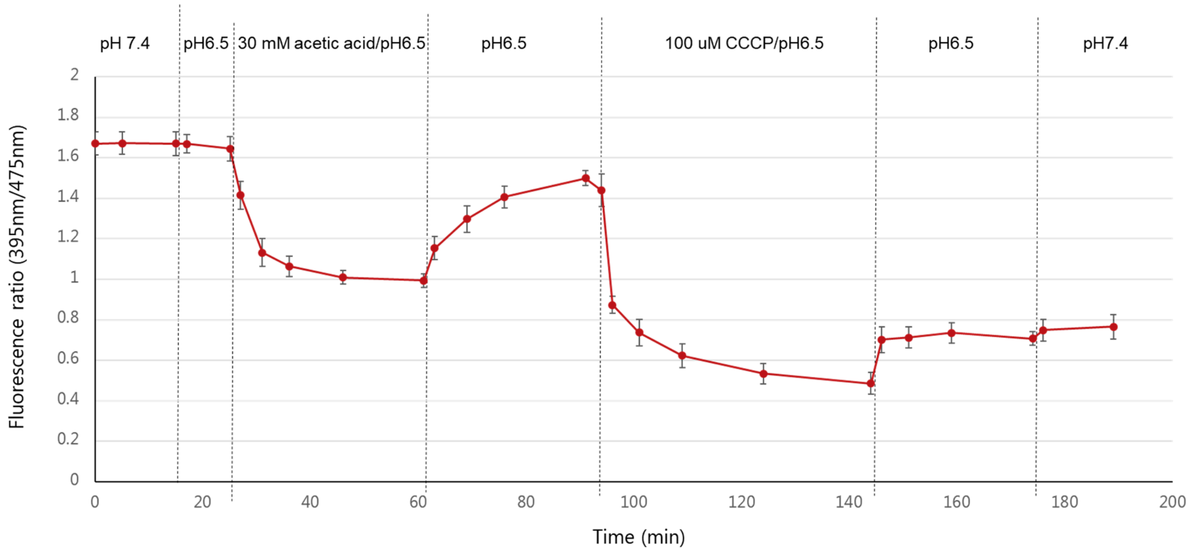

Figure S1. pHlourin2 ratio indicating cytosolic acidification by acetic acid or CCCP. pHluorin2 expressing oocytes. Intracellular acidification of *Xenopus* oocytes by 30 mM acetic acid followed by CCCP – buffers applied to the oocytes on top of the graph. The ratio between the emission at 395 and 475 nm (395/475 nm) represents the relative intracellular pH. Relative cytosolic pH is represented as mean (dot), error bars represent ± s.d

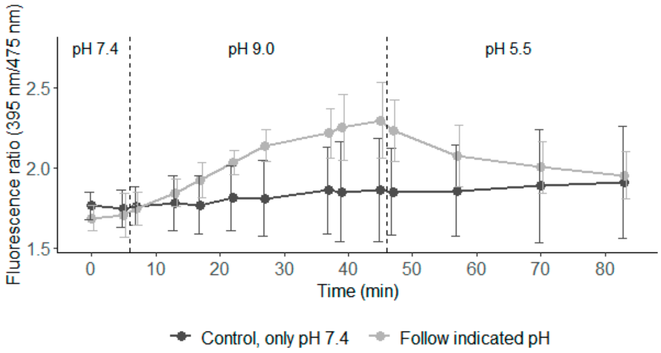

Figure S2. Intracellular alkalization of *Xenopus* oocytes by application of buffer with pH 9.0. pHluorin2 expressing oocytes. Dark grey line represent control oocytes that have had applied only buffer with pH 7.4, while light grey represent oocytes that have had buffers of the specified pH (7.4, 9.0, 5.5) applied. The ratio between the emission at 395 and 475 nm (395/475 nm) represents the relative intracellular pH. Relative cytosolic pH is represented as mean (dot), error bars represent ± s.d. n = 4

Table S1. ANOVA table Figure 1, oocyte samples. Model: aov(Concentration_coumaroylagmatine ~pH/RNA), data = subset_oocyte_samples

	Df	Sum sq	Mean sq	F value	Pr(>F)	
pH	2	15436	7718	29.59	2.51E-08	***
pH:RNA	3	21120	7040	26.99	2.53E-09	***
Residuals	36	9392	261			

Signif. codes: 0 '***' 0.001 '**' 0.01 '*' 0.05 '.' 0.1 ' ' 1

Table S2. ANOVA table Figure 1, buffer samples. Model: aov(Concentration_coumaroylagmatine ~pH/RNA), data = subset_buffer_samples

	Df	Sum sq	Mean sq	F value	Pr(>F)	
pH	1	6099	6099	128.5	5.65E-12	***

pH:RNA	2	18746	9373	197.4	< 2e-16	***
Residuals	28	1329	47			

Signif. codes: 0 '***' 0.001 '**' 0.01 '*' 0.05 '.' 0.1 ' ' 1

Table S3. ANOVA table Figure 3b. Model: aov(Concentration_coumaroylagmatine ~pH/RNA), data = all_data_import

	**Df**	**Sum Sq**	**Mean Sq**	**F value**	**Pr(>F)**	
pH	4	23808	5952	87.64	<2e-16	***
pH:RNA	5	70221	14044	206.78	<2e-16	***
Residuals	58	3939	68			

Signif. codes: 0 '***' 0.001 '**' 0.01 '*' 0.05 '.' 0.1 ' ' 1
